# Chronic silencing of Drd1a-Cre+ neurons impairs dopaminergic-driven cortical activation

**DOI:** 10.3389/fnana.2025.1548545

**Published:** 2025-04-28

**Authors:** Luis Fernando Messore, Auguste Vadisiute, Hayley Edmead, Aleisha Durmaz, Mohammed Abuelem, Flore Chedotal, Anna Hoerder-Suabedissen, Edward Oliver Mann, Zoltán Molnár

**Affiliations:** ^1^Department of Physiology, Anatomy and Genetics, University of Oxford, Oxford, United Kingdom; ^2^St John’s College, University of Oxford, Oxford, United Kingdom; ^3^National Institute of Mental Health, National Institutes of Health, Bethesda, MD, United States; ^4^AgroParisTech, Université Paris-Saclay, Palaiseau, France; ^5^Kavli Institute for Nanoscience Discovery, Sleep and Circadian Neuroscience Institute, Kavli Institute for Nanoscience Discovery, Sleep and Circadian Neuroscience Institute, University of Oxford, Oxford, United Kingdom; ^6^Sleep and Circadian Neuroscience Institute, University of Oxford, Oxford, United Kingdom; ^7^St Hugh’s College, University of Oxford, Oxford, United Kingdom

**Keywords:** dopamine, somatosensory, layer 6b, D1 receptor, subplate

## Abstract

In the somatosensory cortex of transgenic mice, Cre-recombinase is expressed under the control of the dopamine receptor D1 (Drd1a) promoter in lower layer 6. These neurons selectively project to the higher-order thalamic nuclei and participate in the cortico-thalamo-cortical loops involved in sensory processing and stimulus representation. However, the role of dopaminergic modulation in activating this neuronal population during cortical arousal remains poorly understood. In this study, we examined the effects of D1 (SKF-81297) and D2 (Quinpirole) receptor agonists on cortical network activation. We further investigated the consequences of silencing these neurons using a Snap25 conditional knockout mouse model. We report a decrease in cellular and neuronal density in the subplate/L6b with normal development from P8 to adulthood. Conversely, the density of Drd1a-Cre+ neurons goes up in Snap25 cKO brains when comparing the same ages. Moreover, we observe that silencing of Drd1a-Cre+ neurons has no effect on microglial cells. Our results demonstrate that both D1 and D2 agonists require the Drd1a-Cre+ neurons to modulate cortical activity effectively. Our study provides new insights into the fundamental role of Drd1a-Cre+ neurons in cortical activation and sensory processing.

## Introduction

In the murine primary somatosensory cortex (S1), cortical layer 6 neurons play a critical role in cortical arousal via the higher-order corticothalamic system. Among these, the deepest of neurons form a thin compact layer 6b (L6b). In rodents, L6b is considered a remnant of the transient developmental subplate, which is essential for establishing and functionally maturing thalamocortical connections (Ghosh et al., 1990; Kanold and Luhmann, 2010; McConnell et al., 1989). Anatomically, L6b is generally defined as the bottom 30% of L6 or the region within 100 μm of the underlying white matter (Feldmeyer, 2023; Hay et al., 2015; Marx and Feldmeyer, 2013; Zolnik et al., 2024). Laminar-specific and subplate genetic markers such as connective tissue growth factor (Ctgf) and complexin-3 (Cplx3) have also been used to delineate L6b (Arimatsu et al., 2003; Heuer et al., 2003; Hoerder-Suabedissen and Molnar, 2013; Hoerder-Suabedissen et al., 2009). Two L6b subpopulations in S1 have been characterized using specific Cre driver lines: Drd1a-Cre+ neurons expressing Cre recombinase under the promoter of the dopamine D1 receptor, and Ctgf-dgCre+ neurons expressing Cre recombinase under the promoter of the connective tissue growth factor during postnatal development to adulthood (Hoerder-Suabedissen et al., 2018; Messore et al., 2024; Zolnik et al., 2024; Zolnik et al., 2020). In this study, we focused on the Drd1a-Cre+ subpopulation due to its projections selectively targeting higher-order nuclei within the thalamus (Hoerder-Suabedissen et al., 2018). Although ¾ of Drd1a neurons lie within 100 μm of the white matter, there are sparse Drd1a-Cre+ neurons in deep layer 6a (Ledderose et al., 2023). Nevertheless, given their similar physiology and projection patterns, we considered L6b-Drd1 and lower-L6a-Drd1 neurons a homologous group. Unlike L6a-Ntsr1-Cre projection neurons that target both primary and higher-order thalamic nuclei and extend collaterals into the thalamic reticular nucleus (TRN), Drd1a-Cre+ projection neurons exclusively target higher-order thalamic nuclei such as the posteromedial nucleus (POm) without TRN collaterals (Grant et al., 2016; Hoerder-Suabedissen et al., 2018; Olsen et al., 2012). Furthermore, Drd1a-Cre+ synaptic boutons in the POm are smaller compared to L5-Rbp4-Cre corticothalamic terminals (Hoerder-Suabedissen et al., 2018). This suggests that L6b corticothalamic projections modulate higher-order thalamic nuclei in contrast to the large feed-forward “driver” inputs from L5 (Sherman and Guillery, 2002; Zolnik et al., 2024). L6b projections thus appear to regulate thalamocortical circuit activity and transthalamic cortico-cortical communication, processes critical for integrating sensory input with higher cognitive functions (Molnar, 2019).

In S1, Drd1a-Cre+ neurons receive local excitatory input from all cortical layers, most prominently L6a, L5, and L2/3, along with inhibitory input mostly from L6a (Zolnik et al., 2020). Their long-range input is predominately intracortical, primarily from L5 in the ipsilateral motor cortex and L6a in the contralateral S1 (Zolnik et al., 2020). Drd1a-Cre+ neuron projections form two subcircuits in S1: pyramidal projections to L5 and the POm, and non-pyramidal projections targeting L1a apical tuft dendrites of L5 corticothalamic neurons where they induce NMDA-dependent dendritic spikes (Zolnik et al., 2024). Interestingly, Drd1a-Cre+ neurons not only project to and synaptically facilitate higher-order thalamic nuclei like the POm but also functionally excite the cortico-thalamo-cortical loop between L5 and the POm in a topographic manner (Zolnik et al., 2024). Consequently, these Drd1a-Cre+ neurons in S1 play a pivotal role in the bottom-up modulation of cortical activation through the higher-order thalamocortical system.

Although the structural and functional connectivity of Drd1a-Cre+ neurons, as well as their unique response to the wake-promoting neuropeptide orexin, has been extensively studied in S1 (Hoerder-Suabedissen et al., 2018; Zolnik et al., 2024; Zolnik et al., 2020), their dopaminergic modulation remains poorly understood. Dopamine, a monoamine neurotransmitter, mediates diverse functions in the brain, including sensory processing, motor control and higher cognitive functions (Girault and Greengard, 2004). Most dopaminergic projections to the neocortex arise from the ventral tegmental area (VTA) and substantia nigra, forming the mesocortical pathway. G protein-coupled dopamine receptors are divided into five subtypes (D1–D5) that are grouped into two classes: D1-like receptors (D1 and D5) and D2-like receptors (D2, D3, and D4). D1-like receptors are G_s_-coupled receptors that stimulate cyclic adenosine monophosphate (cAMP) production and protein kinase A (PKA) activation, which phosphorylates voltage-gated sodium, potassium, and calcium channels, along with activating the dopamine and cAMP-regulated phosphoprotein DARPP-32 (Di Domenico and Mapelli, 2023). On the other hand, D2-like receptors are G_i/o_-coupled receptors that inhibit cAMP production and PKA activity. D2-like receptors also modulate phospholipase C (PLC) activity that activates protein kinase C (PKC) and phosphatase 2B (PP2B) that inhibits DARPP-32 signaling (Tritsch and Sabatini, 2012). D2-like receptors can also directly activate inward-rectifying potassium channels and inhibit L-type and N-type voltage-gated calcium channels, which further amplifies their inhibitory effects. Both D1-like and D2-like receptors can also modulate GABA, NMDA, and AMPA receptors (Di Domenico and Mapelli, 2023).

Dopaminergic modulation of L6b slow-bursting non-pyramidal neurons in S1 has been demonstrated (Wenger Combremont et al., 2016), and transcriptomic analyses confirm that the D1 receptor is highly expressed in a subtype of cortical L6b neurons (Allen-Brain-Institute, 2020). However, the role of dopaminergic modulation in somatosensory network activity mediated by L6-Drd1a-Cre+ neurons remains unexplored. To address this, we employed a Drd1a-Cre+ functional knockout of the soluble N-ethylmaleimide fusion protein attachment protein receptor (SNARE) complex synaptosomal-associated protein of 25 kDa (Snap25) to chronically “silence” these neurons (Hoerder-Suabedissen et al., 2019). Snap25 is essential for action potential-evoked neurotransmitter release (Bronk et al., 2007; Vadisiute et al., 2022; Washbourne et al., 2002). While global homozygous knockout of Snap25 is lethal, subtype-specific Snap25 knockout from Drd1a-Cre+ neurons across the cortical mantle has been validated to reduce evoked neurotransmitter release while preserving normal initial axonal development and synapse formation (Hoerder-Suabedissen et al., 2019; Molnar et al., 2002). This study investigates the effects of chronically “silencing” corticothalamic Drd1a-Cre+ neurons on cortical neuronal and non-neuronal distribution and dopaminergic modulation of *in-vitro* network activity in the mouse somatosensory cortex.

## Materials and methods

### Animals

All procedures comply with the UK Animals (Scientific Procedures) Act, 1986 (ASPA) and the University of Oxford’s Policy on the Use of Animals in Scientific Research (PP0546018, P828B64BC). All procedures were approved by the University of Oxford Animal Welfare and Ethical Review Board. Animals were held in the Biomedical Sciences Building, Oxford, within individually ventilated cages on a 12-h light/dark cycle. Food and water were supplied ad libitum by the colony manager. To obtain a conditional ablation of Snap25 expression from a subpopulation of cortex layer 6b (Drd1a-Cre Snap25^fl/fl^ cKO) animals B6-Snap25tm3mcw (Snap25^fl/fl^) were crossed to B6;129S6-Gt(ROSA)26Sortm14(CAG-tdTomato)Hez/J (Ai14) and they were crossed to a mouse expressing Cre recombinase Tg(Drd1a-Cre)FK164Gsat/Mmucd (Drd1a-Cre; MMRRC). Experimental mice were produced by crossing female breeders Cre^/+^;Snap25^fl/+^;Ai14^+/+^ (Drd1-Cre+) with male breeders Snap25^fl/fl^;Ai14^+/+^. For experiments comparing control and cKO animals, mice of the following genotypes and abbreviations were used: Drd1a-Cre^−/−^;Ai14^+/+^;Snap25^fl/fl^ (control) and Drd1a-Cre^/+^;Ai14^+/+^;Snap25^fl/fl^ (Snap25 cKO). All lines used were on C57BL/6 background. All genotyping was performed by Transnetyx. Immunohistochemistry, imaging, and analysis were carried out blind to the animal genotype and condition. A more detailed account of the crossing and genotypes can be found in Table 1. For immunohistochemistry, a total of 6 mice were used in this study (*n* = 3 control male and *n* = 3 Snap25 cKO male). For electrophysiology, a total of 8 mice were used for this study [*n* = 5 control (3 female and 2 male) and *n* = 3 Snap25 cKO (2 female and 1 male)].

**Table 1:** Mouse lines crossed in the breeding of the Drd1a-Cre^/+^;Ai14^+/+^;Snap25^fl/fl^ mouse line.

Genotype	Description	References
*Drd1a-Cre; MmRRC*	Tg(Drd1a-Cre)FK164Gsat/Mmucd is a mouse line which selectively expresses Cre-recombinase from the *Drd1a* locus within Layer 6b across the entire cortical mantle from the postnatal period onwards. Cre recombinase expression is under the transcriptional control of the promoter for dopamine receptor D1a. There are several Drd1a-Cre mouse lines with differential expression patterns, in the founder line FK164 Cre recombination is restricted to layer 6b neurons, however the Drd1a-Cre FK164 driver line targets only a partial subset of Drd1a-expressing neurons. Genetic alterations were generated using bacterial artificial chromosome (BAC) engineering. The BAC vector inserted an intron containing cre cassettes and an immediate termination polyadenylation sequence following the recombinase gene	Hoerder-Suabedissen et al. (2018) and Gong et al. (2007), and (GENSAT Brain Atlas), (MMRC)
*Ai14*	The B6;129S6-Gt(ROSA)26Sortm14(CAG-tdTomato)Hze/J (Ai14) conditional reporter line mice express fluorescence labeling in a Cre-dependent fashion. The line utilizes the Rosa26 locus with the ubiquitous CAG promoter followed by the fluorescence gene tdTomato floxed-Stop cassette-controlled allowing for Cre-recombinase dependent transcription of tdTomato	Hoerder-Suabedissen et al. (2019) and Madisen et al. (2010)
*Snap25^fl/fl^*	B6-Snap25tm3mcw mice contain LoxP sites flanking exons 5a and 5b, which in the presence of Cre-recombinase, causes Snap25^fl/fl^ recombination to a truncated transcript. Therefore, *Snap25^fl/fl^* selectively ablates Snap25 protein expression in a Cre-dependent manner	Hoerder-Suabedissen et al. (2019)

### Brain slicing

Mice used for immunohistochemistry analysis were perfused at postnatal day 8 (P8) and P35. Anesthesia of 0.6 mL/kg pentobarbital was administered by intraperitoneal injection (IP) and confirmed via pedal reflex test prior to the perfusion. 0.1 M phosphate buffered saline (PBS, pH 7.4, 79382-50TAB, Sigma Aldrich) and 4% formaldehyde (PFA diluted in PBS, F8775, Sigma) was used to transcardially perfuse the animals. Brains were then removed and post-fixed in 4% PFA overnight at 4°C. The following day, perfused brains were transferred to 1X PBS with 0.05% sodium azide (26628-22-8; Sigma) and stored at 4°C. Brains were then embedded in 4.5% molecular grade agarose (Meridian Bioscience, BIO-41025) and sectioned coronally on a vibrating microtome (Leica, VT1000S) at 50 μm thickness with speed 9 and frequency 7. Slices were then stored in 0.1 M PBS with 0.05% sodium azide (26628-22-8, Sigma) in 24-well plates (Costar).

Mice used for electrophysiology were anesthetized using 4% isoflurane followed by decapitation, and the brains were extracted in cold sucrose solution (40 mM NaCl, 3 mM KCl, 7.4 mM MgSO_4_·7H_2_O, 150 mM sucrose, 1 mM CaCl_2_, 1.25 mM NaH_2_PO_4_, 25 mM NaHCO_3_, and 15 mM glucose; osmolality 300 ± 10 mOsmol/kg). Coronal cortical slices (250–300 μm thick) were cut using a vibratome (Leica VT1200S) and placed in an interface chamber containing artificial cerebrospinal fluid (aCSF) (126 mM NaCl, 3.5 mM KCl, 2 mM MgSO_4_·7H_2_O, 1.25 mM NaH_2_PO_4_, 24 mM NaHCO_3_, 2 mM CaCl_2_, and 10 mM glucose; osmolality 300 ± 10 mOsmol/kg) for the duration of the experiment. All solutions were bubbled with carbogen gas (95% O2/ 5% CO2).

### Immunohistochemistry

Tissue was selected between bregma regions −0.46 mm and −1.46 mm to include the primary somatosensory cortex (S1) region. All sections were blocked with 0.1% Triton-X100 and 2% normal donkey serum (D9663, Sigma) in 0.1 M PBS at room temperature (RT) for 2 h. Sections were then incubated with the primary antibodies in blocking solution at 4°C overnight (Table 2). To determine subplate/L6b cell subpopulation densities, sections were stained with subplate marker rabbit anti-complexin-3 (Cplx3), and to differentiate neurons from other cell types, sections were stained with mouse anti-neuronal nuclear protein (NeuN). To assess microglial density, sections were stained with goat anti-Ionized calcium-binding adaptor molecule 1 (Iba1) or rabbit anti-Iba1. All antibodies have well reported specificity and are commonly used. After washing 3 times with PBS, fluorescent labeling was achieved through incubation with the secondary antibody at RT for 2 h. To determine cells and apoptotic nuclear morphology, all sections were counterstained with DAPI (1:1,000, D1306, Invitrogen). All sections were mounted on Superfrost glass slides and coverslipped in FluorSave^™^ Reagent (345789, Millipore) for imaging. A complete table of the antibodies used can be found in Table 2.

**Table 2:** Summary of antibodies used.

Primary antibodies			
Target	Host species	Concentration	Manufacturer and code
Cplx3	Rabbit	1:1,000	122302, Synaptic Systems
NeuN	Mouse	1:300	MAB377, Sigma-Aldrich
Iba1	Goat	1:300	ab5076, Abcam
Iba1	Rabbit	1:500	019-19741 FUJIFILM Wako

Secondary antibodies			
Fluorophore	Host species	Concentration	Manufacturer and code
Alexa Fluor^®^ 488	Donkey α-Rabbit IgG	1:300	A-21206, Invitrogen
Alexa Fluor^®^ 647	Donkey α-Mouse IgG	1:300	A-31571, Invitrogen
Alexa Fluor^®^ 647	Donkey α-Goat IgG	1:300	A32849, Invitrogen

### Imaging

For the analysis of whole tissue slices (*n* = 3–10 sections/animal), a Thermo Fisher Invitrogen EVOS FL Auto 2 Fluorescent microscope was used to image DAPI labeled sections at 10× air objective and 2.64 μm pixel size, image size 28773 × 9781 μm. To evaluate immunolabeled cell density in L6b of S1 (*n* = 12 images/animal) anti-complexin-3 and anti-NeuN immunolabeled sections were systematically documented using a Nikon Eclipse Ti inverted microscope. Images of L6b were taken using the 40× air objective at 0.2303674 μm pixel size and image size 320.67 × 239.58 μm, while images of the S1 cortex were taken using the 10× air objective at 0.9317711 μm pixel size and image size 320.67 × 239.58 μm. Digital images were acquired on the Quorum Velocity software (version 7.0.0) using a QIClick Camera (01-QIClick-R-F-M-12-C, Qimaging). To examine changes in microglial density and distribution across S1 cortical layers (*n* = 3 images/animal), anti-Iba1 immunolabeled sections were imaged using 2 × 3–4 tiles of images at ×20 air objective and 1 optical zoom at 0.8302662 μm pixel size, tile image size 512 × 512 pixels, using a laser-scanning confocal microscope (Zeiss LSM710).

### Image analysis

Image processing was conducted using open-source software Fiji ImageJ (version 2.9.0/1.53 t). Image filtering to minimize bleed through was semi-automated using macros. Macros were used to assist in semi-automated image preprocessing using ImageJ. Image type was set to 8-bit for faster workflow. Subtract background was used to reduce background noise. Gaussian blur was used for smoothing and noise reduction. Images were displayed as composites to allow for viewing of multiple overlayed channels. Layer area for density analysis was measured using the polygon selection tool. Manual cell counts of complexin-3+ cells (*n* = 3 sections/animal) were obtained from the complexin-3+ and NeuN+ immunolabeled sections. Pre-processed images were uploaded to the image analysis software QuPath (Bankhead et al., 2017).

Automated NeuN+ and tdTom+ cell counts were verified manually. Automated counts of DAPI+, tdTom+, NeuN+, and Iba1+ and DAPI+ copositive cells were obtained using the “positive cell detection” algorithm in QuPath (version 0.4.3) and automated using custom parameters and Groovy scripts. As QuPath cell detection always uses the first channel in a composite image, channels were then split and saved as individual TIF files in a separate folder. Groovy scripts were used for automated cell detection using QuPath. Images type was set to Fluorescent, as all images were immunofluorescent stains. QuPath requires a selected object to work within; as we wanted to detect all cells within an image, we created and set an object as the dimensions of the entire image. We then used custom parameters on the inbuilt cell detection system for each immunofluorescence antibody and image size. The background radius value allows QuPath to estimate background values, then subtract background noise before detection. The median radius microns value allows for texture reduction, allowing for more decreased sigma value use. The sigma value controls Gaussian smoothing and is used to balance the chance of breaking up a cell into multiple positive detections with the chance of blurring multiple cells into one large positive detection. The min and max area values allow for specification of cell size, therefore excluding objects that are too large or small to be considered cells. Lastly, the threshold value is used to define the minimum fluorescence intensity required to consider a positive cell. Lastly, a groovy script was used to export select detection data as a graph on a separate document saved to the project folder.

To evaluate microglial cell density, we created an automatic cell counter program with cell overlapping nuclei [overlap between DAPI (nucleus) and Iba1]. The image processing was done using Python language package Scikit-Image, in particular, the skimage.morphology module to find, filter and count connected volumes. Image filtering operations (Gaussian filter) were performed using skimage.filters module. All scripts can be provided upon request.

### Multi-electrode array recordings

Recordings were done using a high-density microelectrode array (MaxOne) from Maxwell Biosystems (Zürich, Switzerland). The MEA provides 26,400 electrodes in a 3.85 × 2.10 mm^2^ large sensing area with an electrode pitch of 17.5 μm (Muller et al., 2015). A combination of up to 1,024 electrodes can be recorded from simultaneously, with a sampling rate of 20 kHz. All recordings were 5 min long, each condition was done by triplicate. Baseline recordings were done using a continuous administration of 200 μM 4-AP in aCSF for 20 min before recordings started. Afterwards, the solution was changed for a 200 nM Dopaminergic agonist and 200 μM 4-AP in aCSF, following the same protocol as before. The recordings were extracted and pre-processed using custom-made Matlab and Python scripts. All scripts can be provided upon request.

### Statistical analysis

Data are reported as means ± standard error of the mean (SEM). All statistical analysis were done using GraphPad Prism 10. For any single comparison between two groups, we used an unpaired Student’s t-test for normally distributed data, and Mann–Whitney for non-parametric data. Normality was verified using the Shapiro–Wilk test (assessed by QQ plot). For comparison between 3 groups, a one-way ANOVA with Šídák’s multiple comparisons were used. To compare changes in DAPI, NeuN and complexin-3 at P8 and adults between control and Snap25 cKO, we used a two-way ANOVA with Šídák’s multiple comparisons. For laminar distribution of microglial density, a mixed effect model with Šídák’s multiple comparisons was used. Electrophysiological recordings were analysed by mixed effect analysis with Holm–Šídák’s multiple comparisons. The significance level threshold was set at 0.05.

## Results

### Snap25 silencing has no detrimental effect in the normal development of neurite projections to the cortex and to higher order thalamic nuclei

**Figure 1:**
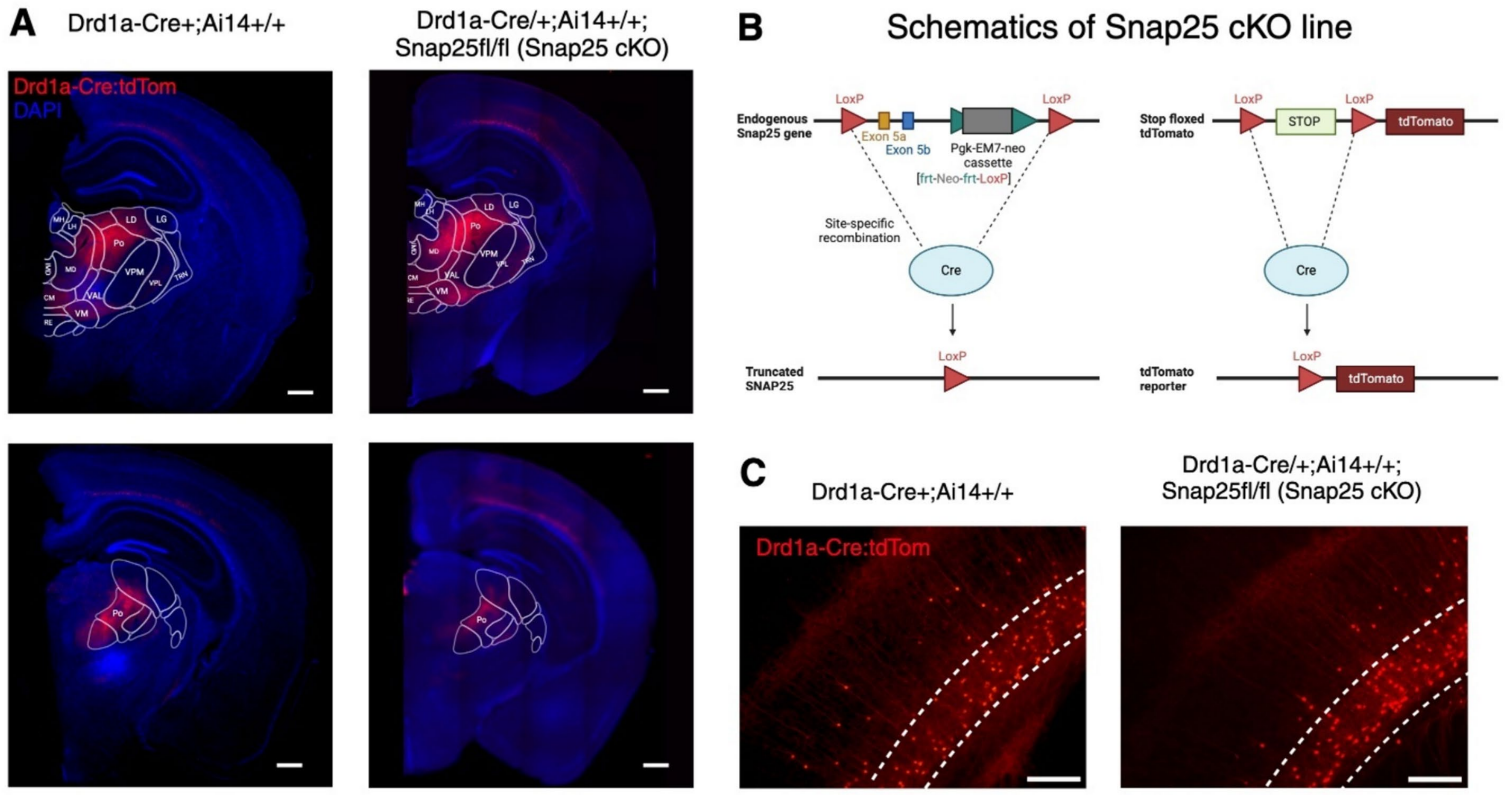
Expression of Drd1a-Cre+ neurons in Snap25 cKO & Drd1a-Cre;Ai14^+/+^ brains. **(A)** Representative images of Drd1a-Cre;Ai14^+/+^ reporter line (left) (Hoerder-Suabedissen et al., 2018) & silenced Drd1a-Cre^/+^;Ai14^+/+^;Snap25^fl/fl^ (Snap25 cKO) line (right). There was no observed difference between the number or distribution of Drd1a-Cre+ neurons (red) between silenced and non-silenced strains. **(B)** Schematics of Snap25 cKO lines (Drd1a-Cre^/+^;Ai14^+/+^;Snap25^fl/fl^). **(C)** Zoom-in representative images of Drd1a-Cre+ neurons in S1. Again, no noticeable difference between the number of Drd1a-Cre+ neurons in either strain was observed. Scale bars of **A**—500 μm, **C**—higher magnification 50 μm. Created with BioRender.com.

To study the effects of chronically abolishing activity-dependent synaptic vesicle release in a subset of layer 6 neurons from birth, we investigated the Drd1a-Cre^/+^;Ai14^+/+^;Snap25^fl/fl^ (Snap25 cKO) mouse line (Figure 1B). As B6-Snap25tm3mcw was used in the generation of the Drd1a-Cre;Snap25^fl/fl^;Ai14^+/+^ line, the validations of Hoerder-Suabedissen et al. (2018) apply to the Drd1a-Cre;Snap25^fl/fl^;Ai14^+/+^ transgenic line. Since tdTomato expression starts from birth (Hoerder-Suabedissen et al., 2018), the Cre recombination is assumed to start from perinatal stages, and Snap25 ablation is from birth. However, spontaneous synaptic vesicle fusion independent of Snap25 function is unaffected by the Snap25 cKO (Washbourne et al., 2002). To assure that the silencing of Drd1a-Cre+ neurons had no measurable effect on the number of Td-Tomato positive neurons, nor in the axonal distribution of these neurons, we compared the otherwise wildtype, Drd1a-Cre+;Ai14^+/+^ line (Hoerder-Suabedissen et al., 2018) with Snap25 cKO animals (Figures 1A,C).

**Figure 2:**
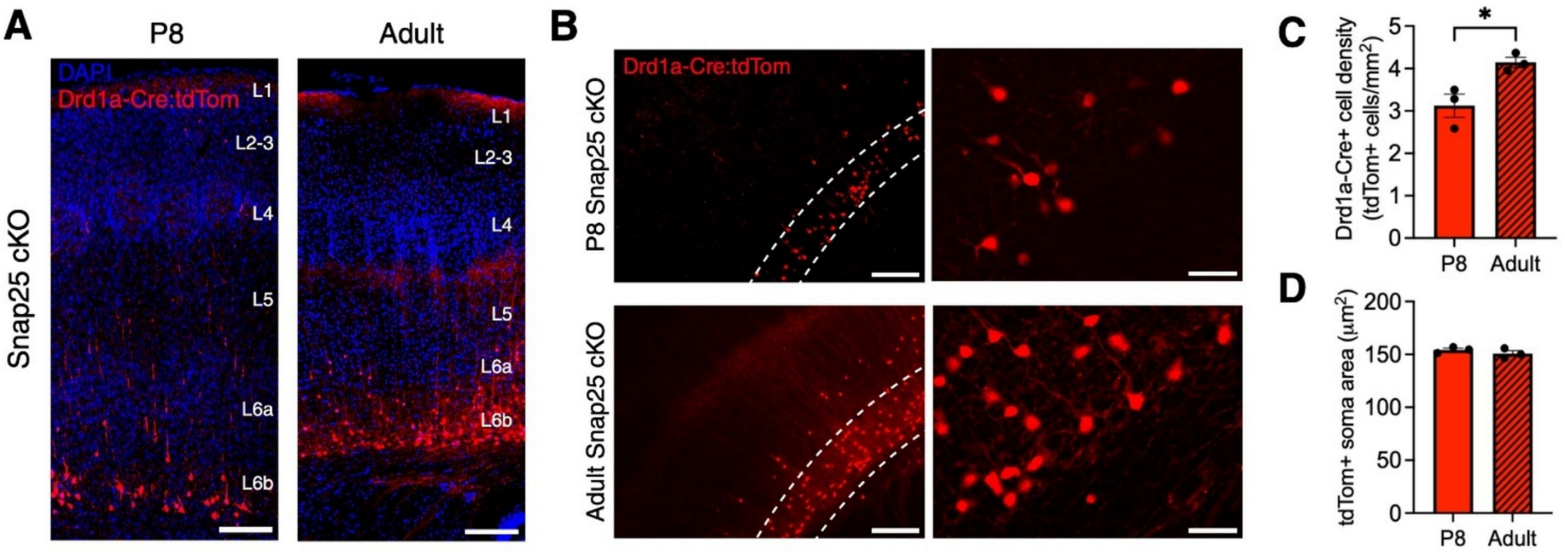
Changes in Drd1a-Cre+ neuron density in developing and adult Snap25 cKO brains. **(A)** Representative images of Drd1a-Cre+ (red) projection neurons in primary somatosensory cortex (S1) in developing (P8) and adult mice. **(B)** Zoom-in representative images of Drd1a-Cre+ neurons in S1. Dotted lines symbolize L6b. **(C,D)** Changes in Drd1a-Cre+ cell density and soma area in Snap25 cKO mice S1 at P8 (*n* = 3) and the adult brains (*n* = 3), with an average of 3 sections per region, evaluated using Student’s *t*-test. All data presented as mean ± SEM, ^*^p < 0.05, ^**^p < 0.01, ^***^p < 0.001, and ^****^p < 0.0001 with scale bars of **A**—500 μm, **C**—200 μm, **D**—lower magnification 20 μm and higher magnification 50 μm. Created with BioRender.com.

To assess the effects of Drd1a-Cre-specific silencing in S1 across time, we measured Drd1a-Cre+ neuron density and soma area in developing (P8) and adult animals. At P8, long, mostly unbranched tomato+ neurites project to L5 (Figures 2A,B). In adults, tomato+ neurites extend to just beneath L4, and axons form a dense network between the border of L4 and L5, as well as in the marginal zone (Figures 2A,B). Occasionally, labeled axons extended into the septa between barrels, as reported for Drd1a-Cre+ cells (Hoerder-Suabedissen et al., 2018), complexin-3+ L6b cells (Viswanathan et al., 2017), and mixed L6a and L6b neurites in the Ntsr1-Cre and Golli-*τ*-eGFP lines (Grant et al., 2016; Pinon et al., 2009). At P8, tdTomato+ cells were less abundant, and tdTomato+ projections were less developed than in adults (Figures 2A,B), as found in control Drd1a-Cre+ cells (Hoerder-Suabedissen et al., 2018). Additionally, at P8, tdTomato+ cell bodies were located mostly in L6b and seldom present in L6a. In adults, L6b tdTomato+ cell density increased (Figure 2C, *t* = 3.391, df = 4, *p* = 0.0275), and cells were seldom present in L6a and L5 (Figures 2A,B). tdTomato+ soma size did not change between P8 and adult brains (Figure 2D, *t* = 1.088, df = 4, *p* = 0.3377).

**Figure 3:**
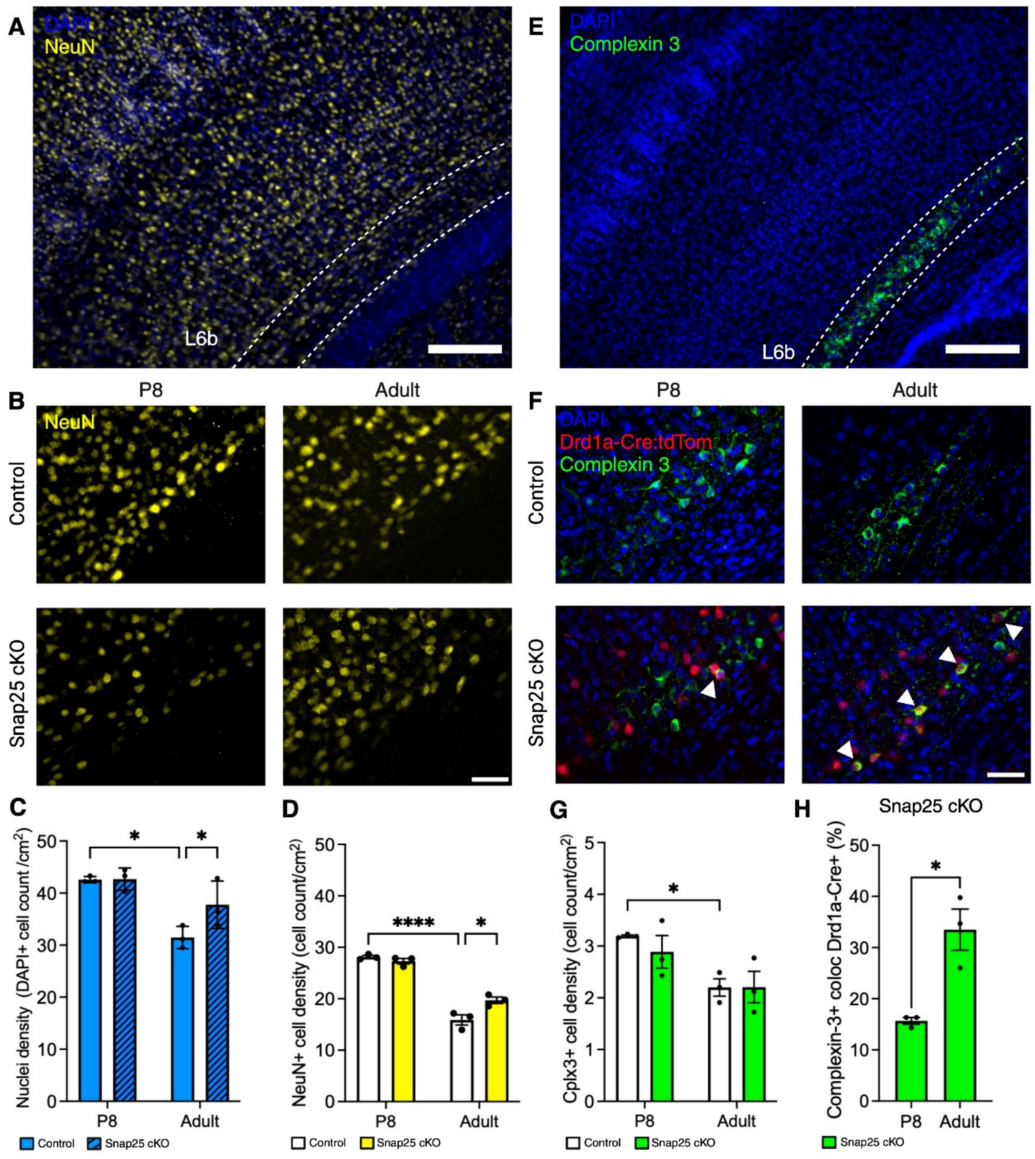
Changes in density and distribution of L6b neurons after synaptic silencing of Drd1a-Cre+ population. **(A)** Representative images of DAPI (blue) and NeuN positive neurons (yellow) in S1 of Snap25 cKO animals. **(B)** Representative images of NeuN (yellow) control and Snap25 cKO at P8 and adult. **(C,D)** Nuclei (blue) and neuron (yellow) densities decrease between P8 and adult in control brains, but not Snap25 cKO brains. Nuclei and neuron density is increased in adult Snap25 cKO brains compared to controls, *n* = 3 (P8) and *n* = 3 (adult) with an average of 3 sections per region, evaluated using two-way ANOVA via Šidak’s multiple comparison. **(E)** Representative images of DAPI positive nuclei (blue) and complexin 3+ cells (green) in S1 of Snap25 cKO animals. **(F)** Representative images of co-localisation between complexin-3 (green) and Drd1a-Cre+ (red) in Snap25 cKO at P8 and adult. **(G)** Complexin-3 (green) cell density decreases between P8 and adult in control brains, but not Snap25 cKO brains. There is no significant difference in complexin 3+ cell density in adult control and Snap25 cKO brains, *n* = 3 (P8) and *n* = 3 (adult) with an average of 3 sections per region, evaluated using two-way ANOVA via Šidak’s multiple comparison. **(H)** Drd1a-Cre+ population in complexin-3+ cells at P8 and adult Snap25 cKO S1 layer 6b, *n* = 3 (P8) and *n* = 3 (adult) with an average of 3 sections per region, evaluated using Mann–Whitney *t*-test. All data presented as mean ± SEM, ^*^p < 0.05, ^**^p < 0.01, ^***^p < 0.001, and ^****^p < 0.0001 with scale bars of **A,E**—200 μm, **B,F**—50 μm. Created with BioRender.com.

We further evaluated neuron density and potential differences in L6b subpopulation, we used immunohistochemical labeling of complexin-3 and co-stained with neuronal marker NeuN (Figures 3A,B,E,F). Complexin-3 is a SNARE complex regulator expressed in high concentrations within layer 6b (Hoerder-Suabedissen et al., 2009). This staining is currently used to anatomically determine the extent of layer 6b in the cortex (Feldmeyer, 2023). First, we quantified DAPI+ nuclei density in S1 L6b. No difference in nuclei density was found at P8. However, in the adult brains, nuclei density was greater in Snap25 cKO than controls. Additionally, nuclei density decreased from P8 to adults in controls, and in Snap25 cKO showed no decrease between P8 and adults (Figure 3C). Furthermore, a similar tendency was observed in NeuN+ cell density, and a statistically significant interaction was observed between age and genotype (*F*_(1,8)_ = 12.25, *p* = 0.0081) and between P8 and adults (*F*_(1,8)_ = 223.4, *p* < 0.0001) but not between genotypes (*F*_(1,8)_ = 5.148, *p* = 0.0530). Moreover, in the control mice, we observed a decrease in NeuN+ cells in the adult S1 L6b compared to P8 (Figure 3D, *p* < 0.0001). In the adult S1 L6b, we observed an increase in NeuN+ cells in Snap25 cKO compared to controls (Figure 3D, *p* = 0.0210).

No differences between Snap25 cKO and control brains were apparent in complexin-3+ cell density at either P8 or adults in S1 (Figure 3G, *F*_(1,8)_ = 0.4036, *p* = 0.5430). However, we observed a decrease in complexin-3+ cell density in control adults compared to P8 (Figures 3G, *p* = 0.0172). Moreover, Drd1a-Cre+ population increased in complexin-3+ cells in the adult brain compared to P8 in Snap25 cKO brains (Figure 3H, *t* = 4.384, df = 4, *p* = 0.0118).

**Figure 4:**
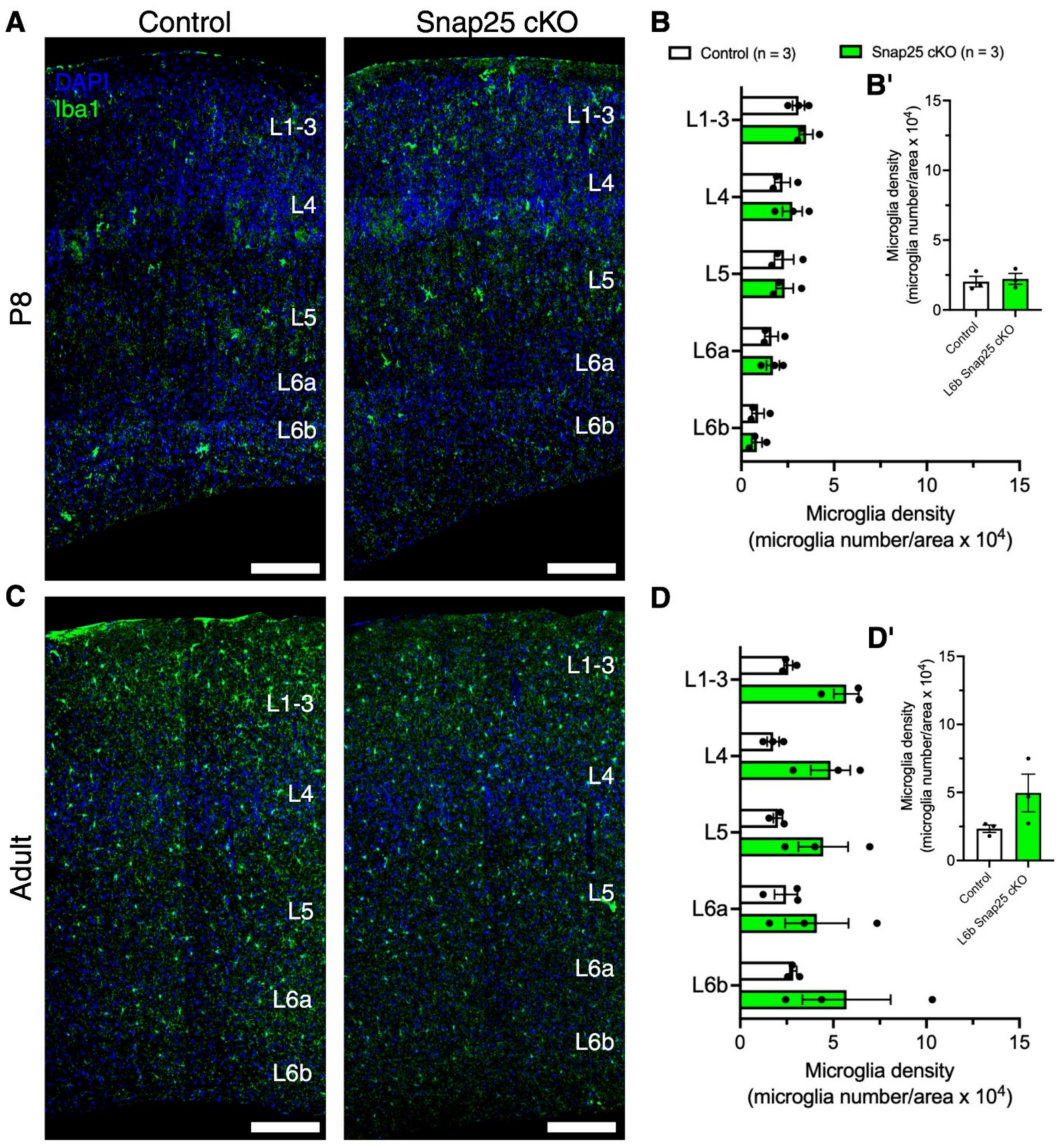
Synaptic silencing of Drd1a-Cre+ neurons has no effect on microglial cells in the primary somatosensory cortex. **(A,C)** Representative images DAPI (blue) positive nuclei and microglial cells (Iba1+, green) in control and Snap25 cKO mice S1 at P8 and adult brains. **(B,B′,D,D′)** There was no difference in microglial cell density between control and Snap25 cKO at P8 and adult mice, *n* = 3 (P8) and *n* = 3 (adult) with an average of 3 sections per region, evaluated using mix-model ANOVA via Šidak’s multiple comparison. All data presented as mean ± SEM, ^*^p < 0.05, ^**^p < 0.01, ^***^p < 0.001, and ^****^p < 0.0001 with scale bars of **A,B**—200 μm. Created with BioRender.com.

As microglia are tissue resident macrophages of the brain, and neuron-microglial communications are activity-dependent, we examined the effect of silencing on microglial cells’ laminar distribution and whole S1 at P8 (Figure 4A) and in adults (Figure 4C). No changes in laminar distribution in microglial density were observed at P8 between control and Snap25 cKO (Figure 4B, *F*_(1,4)_ = 0.1296, *p* = 0.7370) and S1 (Figure 4B′, *t* = 0.3601, df = 4, *p* = 0.7370). Similarly, no changes in laminar distribution in microglial density were observed in adults between control and Snap25 cKO (Figure 4D, *F*_(1,4)_ = 3.477, *p* = 0.1356) and S1 (Figure 4D′, *t* = 1.865, df = 4, *p* = 0.1356).

### Dopaminergic activation of somatosensory cortex

**Figure 5:**
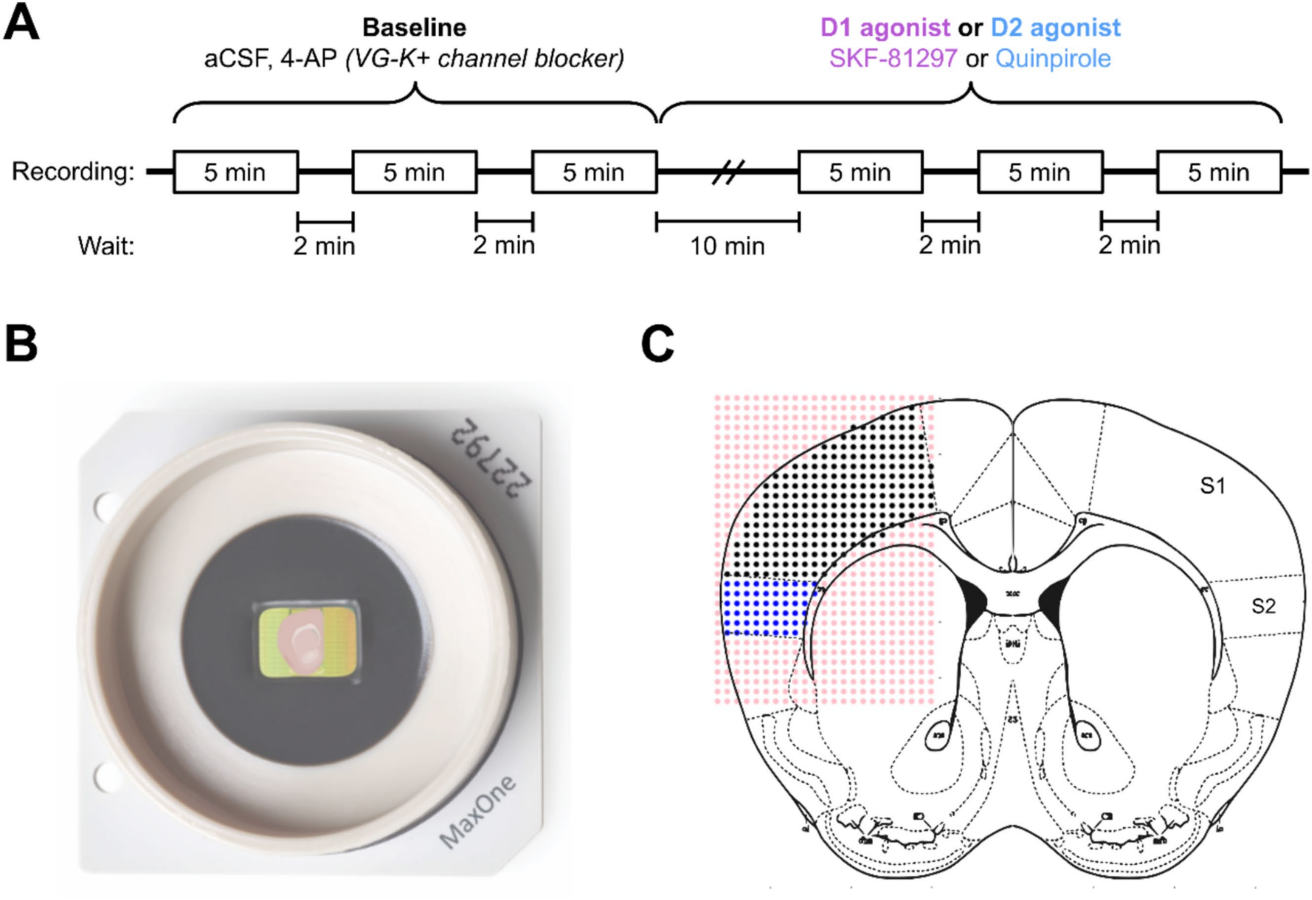
Schematic description of the experimental conditions for electrophysiology. **(A)** Timeline of the electrophysiological recordings in the multielectrode array system. Three baseline recordings with aCSF and a solution of 200 nM of 4-AP were followed by 10 min of a continuous administration of a 2 μM solution of either D1 or D2 agonist. **(B)** Example of the MEA recording system with a slice positioned to ensure that the areas of interest (S1 and S2) contact the recording electrodes. **(C)** Schematic of the labeling of electrodes using the Paxinos mouse brain atlas as a reference.

We evaluated the role of the Drd1a-Cre+ neurons in the maintenance and activation of dopaminergic networks in somatosensory cortex using a multielectrode array (MEA) (MaxOne, Maxwell Biosystems). For the electrophysiological recordings, 300 μm brain slices containing primary somatosensory cortex (S1) and secondary somatosensory cortex (S2) areas were kept in an interface chamber with artificial cerebrospinal fluid (aCSF) at room temperature for at least 2 h before recording. Electrophysiological activity was recorded before and after the administration of a 2 μM solution of either a D1 (SKF-81297) or a D2 (Quinpirole) receptor agonist (Figure 5A). Slices were positioned to ensure that both S1 and S2 areas were in the recording site (Figures 5B,C). The coordinates of the electrodes and the images taken at the time of recording were then mapped onto the Allen Institute mouse brain reference atlas,^1^ resulting in an array with the spatial location of each activity focus and area of interest (S1 and S2). The electrical activity of each electrode was then processed and filtered into two datasets of high-frequency events (spikes) and low-frequency events.

To ensure that the number of Drd1a-Cre+ neurons was similar across animals, only adult mice (157.5 ± 23.97 days) were used. To evaluate if the population of Drd1a-Cre+ neurons drives the dopaminergic activation of the cortical networks, we used the same Snap25 cKO (Drd1a-Cre;Snap25^fl/fl^) genetically modified strain which expresses tdTomato in the Drd1a-Cre+ neurons, as well as having an ablated Snap25 protein (Hoerder-Suabedissen et al., 2019). To start, we measured the effect of the continuous administration of either aCSF or dopaminergic agonists in control animals. The control animals expressed no td-Tomato nor had an ablated Snap25 protein.

**Figure 6:**
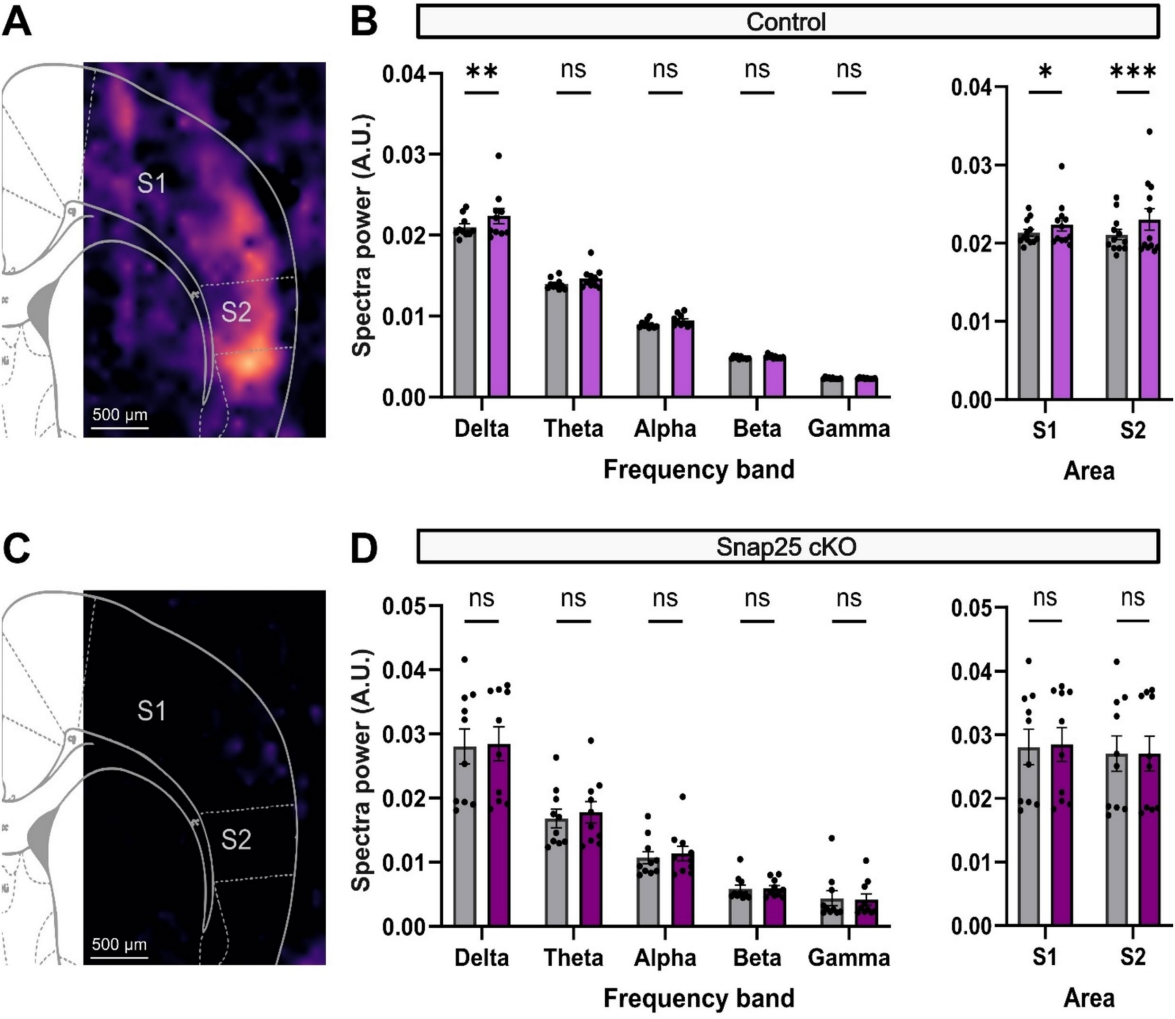
D1 receptor activation of delta frequency band activity in S1 and S2 is impaired by silencing Drd1a-Cre+ neurons. **(A)** PSD analysis of the increase in network response to application of a D1 agonist, 2 μM SKF-81297 solution in the control animals. Warmer colors represent increases in delta power after the drug administration. An increase in activity throughout the cortical mantle can be seen. **(B)** Shows the change in PSD across different brainwaves (Delta − Gamma) before (gray) and after the administration of SKF (magenta). SKF significantly increased the PSD of delta waves in S1. Other brainwaves also showed an increase in PSD, although not significantly. Right panel shows the overall cortical activation in both S1 and S2. Both areas showed a significant increase in activity after SKF administration (*F*_(4,36)_ = 1,009, *p* < 0.0001, mixed-effect two-way ANOVA with repeated measures & multiple comparisons with Šidák correction). **(C)** PSD analysis of network activity after administering 2 μM SKF-81297 solution on Snap25 cKO animals. Silencing of Drd1a-Cre+ neurons disrupts the dopaminergic-driven cortical activation (*p* = 0.0003, two-way ANOVA & multiple comparison with Šidák correction). **(D)** Similarly, panel shows the change in PSD across different brainwaves (Delta − Gamma) before (gray) and after the administration of SKF (purple). Contrasting the previous response, SKF could not significantly increase the PSD of delta waves in S1. Right panel shows the overall cortical activation in both S1 and S2 (*F*_(1,9)_ = 0.5010, *p* = 0.4970, mixed-effect two-way ANOVA with multiple comparisons with Šidák correction). All comparisons were evaluated using mix-model ANOVA via Šidak’s multiple comparison. All data presented as mean ± SEM, ^*^p < 0.05, ^**^p < 0.01, ^***^p < 0.001, and ^****^p < 0.0001.

As it has been shown before (Zolnik et al., 2024; Zolnik et al., 2020), this population both receives and projects to higher order thalamic nuclei, such as POm, and similarly to L5 and L1 through their intracortical projections. Much of the evoked activation from these neurons in the medial prefrontal cortex was observed as an increase in the delta frequency band activity both *in vitro* and *in vivo* (Messore et al., 2024). Here, we showed a similar increase in delta frequency band activity upon administration of D1 receptor agonist (SKF-81297, 2 μM) (Figure 6A). The power spectral density (PSD) in S1 significantly increased from 2.10 × 10^−2^ ± 0.04 × 10^−2^ V^2^/Hz (*n* = 10) to 2.24 × 10^−2^ ± 0.09 × 10^−2^ V^2^/Hz (*n* = 10) after the administration of SKF (Figure 6B). Other frequencies also showed an increase in their respective power densities to the administration of SKF-81297, although these were not significant (Figure 6B). Surprisingly, the activation effect observed was even stronger in S2 which increased from 2.11 × 10^−2^ ± 0.07 × 10^−2^ V^2^/Hz (*n* = 12) to 2.30 × 10^−2^ ± 0.10 × 10^−2^ V^2^/Hz (*n* = 12) (Figure 6B).

To evaluate if this activation is selectively driven by the Drd1a-Cre+ population, we performed a similar set of experiments on Snap25 cKO animals. Chronic silencing of the Drd1a-Cre+ neurons disrupted the D1 receptor agonist-driven cortical activation of S1 and S2 observed in the control animals (Figure 6C). As an example, S1 showed a PSD of delta waves of 2.81 × 10^−2^ ± 0.27 × 10^−2^ V^2^/Hz (*n* = 10) before, and 2.85 × 10^−2^ ± 0.27 × 10^−2^ V^2^/Hz (*n* = 9) after SKF-81297 administration (Figure 6D).

**Figure 7:**
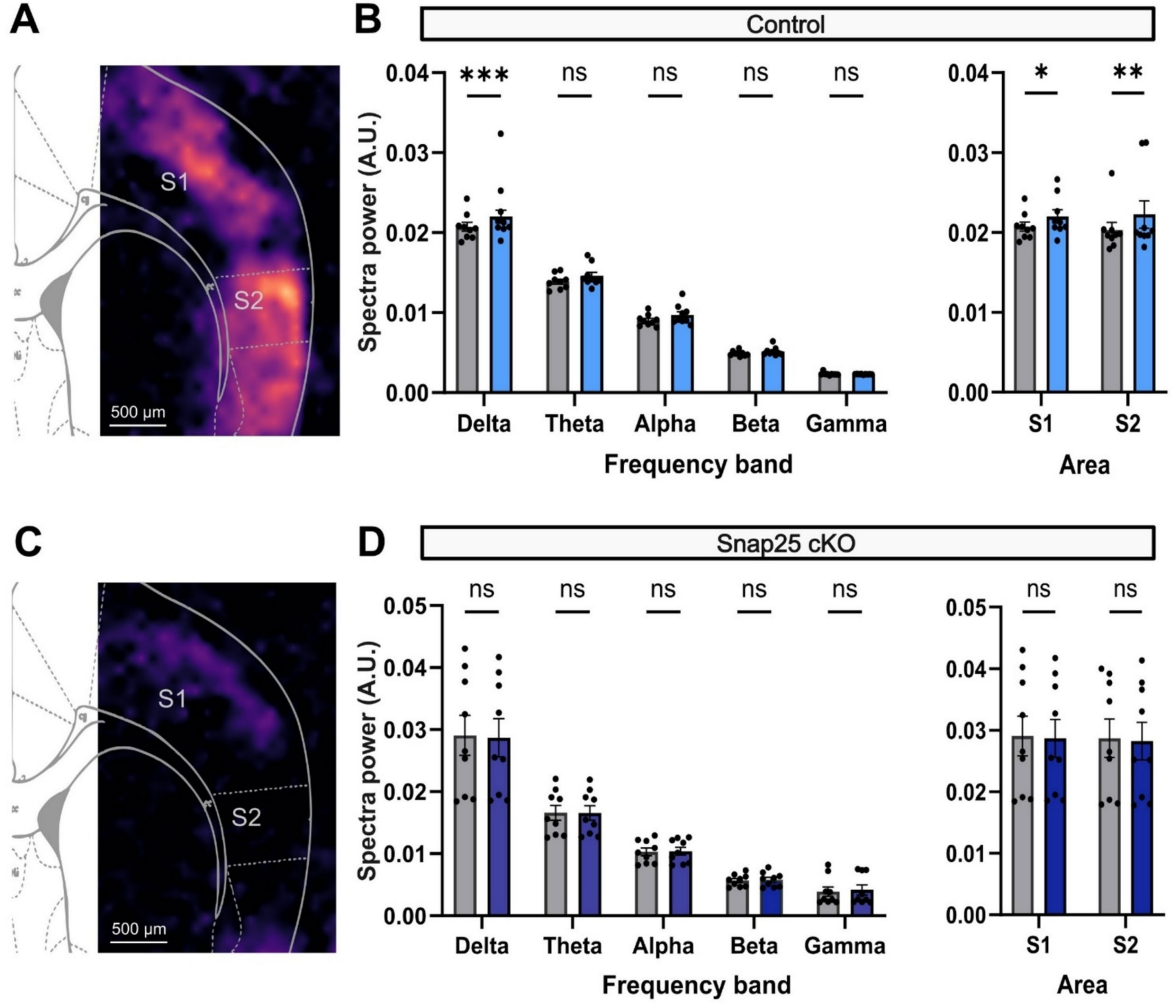
D2 receptor activation of delta frequency band activity in S1 and S2 is impaired by silencing Drd1a-Cre+ neurons. **(A)** PSD analysis of the increase in network response to application of a D2 agonist, 2 μM Quinpirole, in control animals. **(B)** Shows the change in PSD across different brainwaves (Delta − Gamma) before (gray) and after the administration of Quinpirole (light blue). As it was observed for the SKF, Quinpirole significantly increased the PSD of delta waves in S1. Right panel shows the overall cortical activation in both S1 and S2. Both areas showed a significant increase in activity after Quinpirole administration (*F*_(4,32)_ = 842.1, *p* < 0.0001, mixed-effect two-way ANOVA with repeated measures & multiple comparison with Šidák correction). **(C)** PSD analysis of network activity on Snap25 cKO animals. Chronic silencing of Drd1a-Cre+ neurons, again, completely disrupts the dopaminergic-driven cortical activation (*p* = 0.0027, two-way ANOVA & multiple comparisons with Šidák correction). **(D)** Similarly, panel shows the change in PSD across different brainwaves (Delta − Gamma) before (gray) and after the administration of Quinpirole (dark blue). Contrasting with the response in control animals, Quinpirole could not significantly increase the PSD of delta waves in S1. Right panel shows the overall cortical activation in both S1 and S2 (*F*_(1,8)_ = 1.253, *p* = 0.2954, mixed-effect two-way ANOVA with multiple comparisons with Šidák correction). All comparisons were evaluated using mix-model ANOVA via Šidak’s multiple comparison. All data presented as mean ± SEM, ^*^p < 0.05, ^**^p < 0.01, ^***^p < 0.001, and ^****^p < 0.0001.

The activation of the cortical networks by the administration of D2 receptor agonist (Quinpirole) mirrored those for D1 receptor agonist. An increase in delta frequency band activity across the somatosensory areas was observed after the administration of Quinpirole (2 μM). Interestingly, although the activation was observed in the same areas of the cortex, the activation pattern caused by quinpirole seems to be much more general across the cortical mantle (Figure 7A). Nevertheless, the activation was most powerful in the delta waveband where the PSD significantly increased 2.07 × 10^−2^ ± 0.06 × 10^−2^ V^2^/Hz (*n* = 9) to 2.20 × 10^−2^ ± 0.08 × 10^−2^ V^2^/Hz (*n* = 9) after the administration of Quinpirole (Figure 7B). Other frequencies also showed an increase in their respective power densities to the administration of QP, although these were not significant (Figure 7B).

Consistent with the results mentioned above, the D2 receptor activation effect observed was stronger in S2, which increased from 2.03 × 10^−2^ ± 0.09 × 10^−2^ V^2^/Hz (*n* = 9) to 2.23 × 10^−2^ ± 0.17 × 10^−2^ V^2^/Hz (*n* = 9) (Figure 7C). Silencing Drd1a-Cre+ neurons (Snap25 cKO) impaired the D2 receptor-mediated activation of S1 and S2, similar to the effect on D1 receptor agonist evoked activity (Figure 7C). In this case, S1 showed a PSD of delta waves of 2.91 × 10^−2^ ± 0.32 × 10^−2^ V^2^/Hz (*n* = 9) before, and 2.87 × 10^−2^ ± 0.31 × 10^−2^ V^2^/Hz (*n* = 9) (Figure 7D).

## Discussion

We studied the dopaminergic modulation of Drd1a-Cre+ cortical neurons, a population shown to have selective sensitivity to orexin and projections to the cortex and higher-order thalamic nuclei (Case and Broberger, 2018). We genetically silenced these neurons via conditional knock-out (loss of function) of Snap25 shortly after birth, removing their ability to communicate to their cortical and thalamic targets through regulated vesicular release. Our study showed changes in Drd1a-Cre+ cortical neuron density in Snap25 cKO brains between P8 and adult. We further showed that the modulation of cortical activity through D1 and D2 receptor agonists is reduced if the Drd1a-Cre-expressing lower layer 6 neurons are genetically silenced. Our study provides new insights into the fundamental role of Drd1a-Cre+ cortical L6 neurons in cortical activation that could substantially influence the brain state-specific cortico-thalamo-cortical processing.

### Regulated synaptic vesicle release by Drd1a-Cre+ neurons is not required for normal anatomical development

In postnatal mice, Snap25 ablation in a subset of subplate neurons (SPns) had no impact on cytoarchitectural development. It was previously reported that Snap25 KO embryos display histologically normal development of the central nervous system (Molnar et al., 2002; Washbourne et al., 2002). However, the Munc18-1 KO model, as well as other layer-specific Snap25 cKO models Rbp4-Cre;Snap25^fl/fl^ and Ntsr1-Cre;Snap25^fl/fl^, demonstrate neurodegeneration and mature neuron cell death, albeit on a much longer and protracted time scale (Hoerder-Suabedissen et al., 2019; Verhage et al., 2000). As such, synaptic connectivity formation may be independent of regulated neurotransmitter secretion, while maintenance of established synaptic connections may rely on synaptic vesicle release. While the Snap25 cKO line described in this project has been assessed up to 8 months of age (Hoerder-Suabedissen et al., 2019), analysis of brains older than 8 months may demonstrate similar findings of neurodegeneration and degenerative cell death. Moreover, axons from the Drd1a-Cre+ neurons in Snap25 cKO mice showed no significant reduction in the number of Cre+ cell bodies nor thalamic input. Nevertheless, some of those axons appear fragmented and are absent from the thalamus by 8 months of age (Hoerder-Suabedissen et al., 2018). Our findings and other genetic ablation studies are in contrast to the results of early subplate ablation studies. Ablation of SPns through p75-immunotoxin or kainic acid injection in cats brain prevents LG axon segregation in L4 (Ghosh and Shatz, 1992) and disrupts orientation column and ocular dominance column (ODC) formation in rats brain (Kanold et al., 2003), suggesting SPns are required for cortical circuit assembly and for the formation of cytoarchitectural pattern in the barrel field. The contrasting findings between SNARE protein genetic cKO and SPn ablation studies may be reconciled by the potential relevance of other-synaptic communication, such as gap junctions (Dupont et al., 2005) or connexin (cx) hemichannels (Moore et al., 2014). In the rodent neocortex, from P0-P4 gap junction coupling synchronizes electrical activity (Corlew et al., 2004). Additionally, *in vitro* human fetal (17–23 gw) cortex subplate neuron membrane depolarisation measured with whole-cell patch-clamp recording is not completely silenced by pharmacological occlusion of synaptic transmission (Moore et al., 2014). However, application of cx hemichannel blocker lanthanum strongly inhibited the frequency and amplitude of spontaneous electrical activity. On the other hand, as Drd1a-Cre labels 18–24% of S1 SPns (Hoerder-Suabedissen et al., 2018), by only silencing a portion of subplate/L6b neurons, albeit a significant amount, other unaffected SPns projections to cortico-cortical and corticothalamic circuitry may compensate for the Snap25 ablation. Additionally, the onset of Cre expression in cortex of Drd1a-Cre+ mice is from P0. Therefore, SPn activity-dependent synaptic vesicle release occurs as normally across the embryonic period, which may also affect the SPn projection and circuitry development. In conclusion, the establishment of corticothalamic circuits is independent of Drd1a-Cre+ neuronal communication through activity-dependent vesicular release from the time of birth. Specifically, early thalamic axon ingrowth and L6b neurite patterning are each independent processes to activity-dependent regulated synaptic vesicle release from this subpopulation of L6b neurons.

### Silencing Drd1a-Cre+ neurons increases adult somatosensory L6b neuron density

In the adult brains, NeuN+ cell density is only increased in L6b of S1 in Snap25 cKO brains. As this difference in density was not evident at P8, the increase in NeuN+ cells in Snap25 cKO adult L6b may be due to stranded migratory neurons that were originally destined for the cortical plate. Synaptic transmission from SPns is necessary for radial neuron migration from the ventricular zone toward the pial surface through transient glutamatergic synapses in embryonic mouse brains (Ohtaka-Maruyama et al., 2018). The synaptic output from SPns to migrating neurons temporally regulates the change in migration mode of migrating neurons; knockout of postsynaptic proteins PSD-95 and NR1 in migrating neurons resulted in arrested migration of knockout neurons at the subplate zone boundary. As cortical neuron migration continues postnatally in mice (Yoshinaga et al., 2022), the cKO of synaptic input from SPns could also result in arrested neuron migration. While these transient synaptic contacts between SPns and migrating neurons have only been evaluated in embryonic mice, further analysis of postnatal synapse formation between SPns and migrating neurons using similar techniques is required.

On the other hand, during subplate dissolution, neuron density decreases due to apoptosis (Torres-Reveron and Friedlander, 2007). Our results reflect this, as L6b NeuN+ and complexin-3+ densities decrease significantly between P8 and adults in control brains. DAPI nuclei density analysis suggested nuclei concentration to decrease between P8 and adults in ctrl S1 L6b. This decrease was less evident in the adult Snap25 cKO brain. Most neurons in L6b/subplate decreases during development maintaining lower population of neurons in the mature L6b. In L6b, we observed a decrease of NeuN+ neurons in L6b between P8 and adult brain (Hoerder-Suabedissen and Molnar, 2013). We also observed and increase in NeuN+ neurons in adult Snap25 cKO compared to adult control brains. Moreover, Drd1a-Cre+ cell number is higher in Snap25 cKO adults compared to Snap25 cKO P8. We can speculate that increase in Snap25 cKO L6b is due to deletion of Snap25. It might be related with altered synaptic pruning or phagocytic activity of glial cells since communication between neuron and microglial cells is activity-dependent. To better understand if increase in neurons in L6b due to altered synaptic pruning and evaluate glial phagocytic activity more studies need to be performed especially during peak of synaptic pruning at P14/15 (Kim et al., 2015; Paolicelli et al., 2011). It has been shown that the number of Drd1a-Cre+ neurons increase between P1 and P8 and Drd1a-Cre+ population is present in 10% of complexin-3+ cells (Hoerder-Suabedissen et al., 2018). Here, we observed that in Snap25 cKO mice S1 L6b, Drd1a-Cre+ population is present in 33% of complexin-3+ cells in the adults. As neuron migration and apoptosis are both age-specific developmental processes, a better resolution in time points assessed would provide insight into the kinetics and initiation of divergence in NeuN+ neuron density and cell death between the control and Snap25 cKO.

We further investigated microglial cells density and distribution. In the Snap25 cKO brains, no changes in Iba1+ microglial density were observed, suggesting that silencing of Drd1a-Cre+ neurons does not cause inflammatory responses. This is in contrast to other Snap25 silenced mouse lines (i.e., Rbp4-Cre;Snap25^fl/fl^) where microglia rearrangements were observed in layer 5 (Vadisiute et al., 2024).

### Characterization of the Drd1a-Cre+ population in S1

Anatomically, L6b is generally considered a thin compact cytoarchitectonic entity that is separated both from white matter/intermediate zone and layer 6 proper and forms the bottom 30% of L6 or is located within ~100 μm of the underlying white matter (Feldmeyer, 2023; Hay et al., 2015; Marx and Feldmeyer, 2013; Zolnik et al., 2024). Two L6b subpopulations have been characterized using specific Cre driver lines: Drd1a-Cre+ neurons (which express Cre-recombinase through the activation of the dopamine D1 receptor promoter) and Ctgf-dgCre+ neurons (which express Cre-recombinase through the activation of the promoter of connective tissue growth factor from early postnatal stages through adulthood). Drd1a neurons and Ctgf neurons represent 25–40 and 25% of S1 L6b neurons, respectively (Hoerder-Suabedissen et al., 2018; Zolnik et al., 2020). In S1, both L6b subpopulations receive local intracortical excitatory input from all layers, especially L6a, L5, and L2/3, along with inhibitory input mostly from L6a. In primary somatosensory cortex, around 95% of their long-range input is intracortical, including both ipsilateral and contralateral, and they receive little subcortical input, with the most prominent exception being the higher-order posteromedial thalamus (POm) (Zolnik et al., 2020).

The thalamic projections of Drd1a-Cre+ neurons almost exclusively target higher-order nuclei such as the POm (Hoerder-Suabedissen et al., 2018). Furthermore, L6b is the only cortical layer responsive to the wake-promoting neuropeptide orexin (Bayer et al., 2004), and Drd1a-Cre+ neurons are the only projection neurons in L6 that respond to orexin, suggesting that these neurons are likely modulated by orexinergic neurons from the lateral hypothalamus and play an important role in cortical arousal via the higher-order thalamocortical system (Bayer et al., 2004; Messore et al., 2024; Zolnik et al., 2024). Because Drd1a-Cre+ neurons highly express the D1 receptor, their dopaminergic modulation may play a synergistic role with orexin in driving cortical arousal. Given the enhanced D1 receptor expression in L6 (Allen-Brain-Institute, 2020; Berger et al., 1991; Santana et al., 2009), we sought to characterize their electrophysiological response to D1 and D2 receptor modulation in S1.

### Dopaminergic-driven cortical activation of somatosensory cortices

In order to characterize these physiological responses, we administered a 2 μM solution of a D1 receptor agonist (SKF-89127) or a D2 receptor agonist (Quinpirole) to coronal brain slices that contained the somatosensory areas S1 and S2 (Clark and Bracci, 2018; Lancon et al., 2021; Stagkourakis et al., 2016). Both administrations showed an increase in delta frequency band activity in the somatosensory areas of control brains. Mirroring the effects observed for the activation of Drd1a-Cre+ neurons in the prefrontal cortex, dopamine agonists induced an increase in cortical activity, most significant in the low-frequency brainwave bands such as Delta (0.5–4 Hz) and Theta (4–8 Hz) (Harmony, 2013; Messore et al., 2024; Rivera-Lillo et al., 2021). Interestingly, the activation was stronger in S2 compared to S1. Although both areas have a considerable number of Drd1a-Cre+ neurons, their density is generally lower in S2 than in S1. Thus, the proposed role of these neurons as part of the cortico-thalamocortical loops would better explain their strong activation of cortical association areas such as S2. To assess if this activation was driven by the Drd1a-Cre+ population, we repeated these experiments in genetically silenced Drd1a-Cre+ animals (Snap25 cKO). We observed that the activation of both S1 and S2 was impaired regardless of which dopaminergic agonist was administered.

It has been suggested that Drd1a-Cre+ neurons function as a unidirectional activator of the thalamocortical system, notably due to their lack of direct input from the cortico-thalamocortical loop (Zolnik et al., 2024). Our findings suggest that dopamine may serve an important role in facilitating this thalamocortical activation, likely in a synergistic role with orexin (Zolnik et al., 2024; Messore et al., 2024). This dopaminergic activation across S1 and S2 may be differentially mediated through two distinct Drd1a-Cre+ subcircuits: those projecting to cortical L5 and POm, and those projecting to L1a apical tuft dendrites of L5 corticothalamic neurons, which induce NMDA-dependent spikes (Zolnik et al., 2024). Because D1 receptors potentiate NMDA-mediated responses in cortical layer 5 neurons (Zheng et al., 1999; Seamans et al., 2001; Wang and O’Donnell, 2001), D1 agonists may particularly facilitate the latter subcircuit of Drd1a-Cre+ neurons. Overall, our results suggest that the Drd1a-Cre+ population drives the dopaminergic activation of the cortical networks in both primary and secondary somatosensory areas. Moreover, this activation seems to have as one of its primary targets the cortico-thalamocortical loops found in both primary and secondary cortices for stimulus representation and sensory processing.

## Data Availability

The original contributions presented in the study are included in the article/supplementary material, further inquiries can be directed to the corresponding authors.

## Footnotes

^1^ https://atlas.brain-map.org/
